# Emergency holmium laser enucleation of the prostate for management of refractory hematuria

**DOI:** 10.1007/s00345-026-06707-4

**Published:** 2026-08-18

**Authors:** Jack T. Peterson, Jenny Guo, Amir Patel, Jessica Helon, Alyssa McDonald, Allaa Fadl-Alla, Perry Xu, Amy E. Krambeck

**Affiliations:** 1 Department of Urology, Northwestern University Feinberg School of Medicine, 676 N. Saint Clair St., Arkes 2300, Chicago, IL 60611 USA; 2 Department of Urology, Oregon Health and Science University, 10000 Southeast Main Street, Suite 260, Portland, OR 97216 USA

**Keywords:** Holmium laser enucleation of the prostate, Hematuria, Benign prostatic hyperplasia, Cystoscopy

## Abstract

**Purpose:**

To evaluate the safety and efficacy of emergency holmium laser enucleation of the prostate (HoLEP) for the management of refractory hematuria in the acute inpatient setting.

**Methods:**

A retrospective review of patients who underwent emergency HoLEP in the inpatient setting between January 2021 and August 2024 was performed. All procedures were performed by a single surgeon. Clinical and operative variables including indication for surgery, intra-operative findings, operative time, enucleated prostate volume, hospital course, perioperative transfusion requirements, post-operative catheterization status, and complications were collected. Post-operative outcomes including voiding trial success and catheter status at follow-up were assessed. Descriptive statistics were used to summarize patient characteristics and perioperative outcomes.

**Results:**

A total of 24 emergency HoLEPs were performed: all for refractory hematuria and one additionally for prostate abscess. During the same admission, five patients had previously undergone cystoscopic fulguration and evacuation procedures. Eight patients had prior transurethral resections of the prostate, two photoselective vaporization procedures, and one prostate artery embolization. The median operative time was 102 min, and the median enucleated prostate volume was 156 g. Eighteen (75%) of the patients passed their voiding trial on post-operative day 1, and all were catheter-free at three-month follow-up. Ten patients required blood transfusions, including eight preoperatively and seven postoperatively. No patients required additional procedures.

**Conclusion:**

From our cohort, emergency HoLEP appears to be a safe and effective treatment for refractory hematuria. Patients demonstrated high rates of early catheter removal and catheter-free voiding at follow-up, with no patients requiring additional procedures.

## Introduction

Benign prostatic hyperplasia (BPH) is a prevalent condition among aging men that can lead to lower urinary tract symptoms (LUTS), recurrent urinary tract infections, and gross hematuria (GH) [1, 2]. GH is particularly prevalent in BPH patients due to hypervascularity and inflammation in the prostate [3]. In addition, usage of anti-coagulants and anti-platelet agents for treatment of other medical conditions can put patients at higher risk of developing hematuria. While conservative management is often sufficient, a subset of patients develop refractory, potentially life-threatening GH requiring hospitalization for continuous bladder irrigation, blood transfusions, and ultimately surgical intervention [4–6]. Notably, persistent gross hematuria alone frequently prevents safe discharge, resulting in prolonged hospitalization even when patients are otherwise clinically stable. The optimal treatment strategy for refractory GH in the setting of BPH remains elusive, especially in patients with large prostates and those on anticoagulant and antiplatelet therapies [7].

Common surgical approaches for refractory GH include transurethral resection of the prostate (TURP), Greenlight™ photovaporization of the prostate (PVP) and prostatic arterial embolization (PAE) [5, 8, 9]. However, among these modalities, only PAE has been specifically evaluated for gross hematuria secondary to BPH refractory to medical treatment, in which recurrent hematuria was reported in 15% of patients at 3 months and an 80% medium-term clinical success rate at 12 months [10]. American Urological Association (AUA) guidelines recommend holmium laser enucleation of the prostate (HoLEP) as a size-independent treatment option for LUTS/BPH, dependent on clinician expertise [6]. Multiple studies have demonstrated that HoLEP provides superior hemostatic control, lower transfusion rates, and improved perioperative outcomes compared to TURP, even in patients on anticoagulation [11, 12].

Emergency HoLEP is used for urgent, non-elective intervention in the acute inpatient setting for patients with refractory hematuria. These patients often have concurrent infection or retention and have failed conservative management, barring them from safe discharge without definitive intervention. Common clinical scenarios include persistent bleeding despite continuous bladder irrigation (CBI), clot evacuation with or without fulguration, and prior PAE. As such, emergency HoLEP represents a distinct clinical application of HoLEP aimed at achieving rapid and durable hemostasis in a high-risk population.

Despite these advances, the use of HoLEP as an emergency therapy for refractory GH due to BPH is not as well characterized, and data regarding surgical success and post-operative outcomes in this context is limited. This study aims to address the safety and efficacy of HoLEP in an emergency setting, such as in cases of refractory hematuria, large-gland BPH, and concurrent anticoagulant/antiplatelet usage.

## Methods

Under Institutional Review Board approval and in accordance with the ethical standards of the 1964 Helsinki Declaration, we conducted a retrospective review of men with prostate volumes greater than 30 cubic centimeters who underwent HoLEP for refractory GH at our institution between January 2021 to August 2024. All HoLEP procedures were performed by a single surgeon using high-powered pulse-modulated holmium laser technology. In the setting of active bleeding, higher power laser settings (2.0 J × 60 Hz) were preferentially utilized to optimize hemostasis. HoLEP was performed using a continuous flow 28 Fr endoscopic system. The larger caliber endoscope allows for improved visualization in the face of active bleeding and facilitates efficient evacuation of intravesical clot burden. Attention was given to intraoperative assessment of clot burden, with systematic inspection and clearance performed prior to enucleation to optimize hemostasis. Patients were selected for emergency HoLEP after inpatient urology consultation for refractory gross hematuria, typically with clot retention and failure of conservative management. Before enucleation was considered, patients generally underwent pelvic imaging to assess clot burden and prostate size, Foley catheter placement with or without continuous bladder irrigation, manual clot irrigation as needed, and blood transfusion when clinically indicated. The treating urologist determined the need for preoperative cystoscopy as part of the overall clinical decision-making process. Emergency HoLEP was considered when hematuria remained uncontrolled despite these measures and prevented safe discharge or clinical stabilization. All cases were scheduled as urgent inpatient add-on procedures during the index admission, typically performed on the day following clinical stabilization and initial evaluation. Antifibrinolytic agents including aminocaproic acid (Amicar), tranexamic acid, and hydrogen peroxide (H₂O₂) bladder irrigation were not utilized during the study period as part of institutional practice.

Baseline characteristics, intraoperative variables, and post-operative outcomes were collected for each patient. Baseline characteristics included age, body mass index, prostate volume, American Society of Anesthesiologists physical status classification, and anticoagulation or antiplatelet use. Pre-operative clinical course variables were also collected, including the number and type of failed interventions prior to HoLEP. Timing variables included duration from onset of gross hematuria to operative intervention and time from prior failed intervention to HoLEP.

The longitudinal treatment course was documented for each patient, including the sequence of interventions and location of care prior to surgery. Descriptive statistics were used to summarize patient characteristics and perioperative outcomes. Continuous variables are reported as median with interquartile range and categorical variables as counts and percentages. When clinically relevant, ranges are also provided to describe the full observed spread. Statistical analyses were performed using IBM SPSS Statistics version 28.0 (Armonk, NY, USA).

## Results

Patient demographics, pre-operative metrics, and clinical characteristics are detailed in Table 1. A total of 24 emergency HoLEP procedures were performed for refractory GH between January 2021 and August 2024. One procedure was performed for concomitant prostate abscess and refractory hematuria. Notably, all 24 patients presented to the emergency department prior to admission. The initial reason for urology consultation was isolated gross hematuria in 18 (75.0%) patients, hematuria with urinary retention or suprapubic pain in 4 (16.7%), and hematuria with systemic symptoms (i.e. syncope and chest pain) in 2 (8.3%). Thirteen patients had undergone prior cystoscopic procedures, including five who had cystoscopy with fulguration and clot evacuation, and eight who received flexible cystoscopies. Among the 24 patients, ten had a history of prior BPH interventions, including one PAE, two PVP procedures (one being Greenlight™), and eight TURP. All patients had failed at least one prior intervention for hematuria management, with continuous bladder irrigation representing the minimum initial therapy in all cases.

**Table 1:** Participant demographic and preoperative clinical characteristics

Characteristic	*N* = 24
Age (years) ^1^	74 (67.5-77.25)
BMI (kg/m^2^) ^1^	27 (23.7–32)
ASA Class ^2^	
2 ^2^	4 (16.7)
3 ^2^	16 (66.7)
4 ^2^	4 (16.7)
Prostate volume (mL) ^1^	216.5 (144.3-314.3)
Prior BPH procedure ^2^	10 (41.7)
TURP ^2^	8 (33.3)
PVP ^2^	2 (8.3)
PAE ^2^	1 (4.2)
Prior hematuria procedure ^2^	5 (20.8)
Indwelling Foley catheter prior to admission ^2^	15 (62.5)
Admission and presentation context	
Presented from Emergency Department ²	24 (100)
Initial reason for presentation ²	
Isolated gross hematuria	18 (75)
Hematuria with retention/suprapubic pain	4 (16.7)
Hematuria with systemic symptoms	2 (8.3)
On anticoagulants ^2^	11 (45.8)
Held prior to surgery ^2^	10 (41.7)
On antiplatelets ^2^	9 (37.5)
Held prior to surgery ^2^	6 (25)
Pre-operative clinical course	
Hemoglobin before HoLEP (g/dL) ¹	10.6 (8.0–12.5)
Received pre-operative blood transfusion ²	8 (33.3)
Foley catheter placed on index admission ²	9 (37.5)
CBI performed ²	22 (91.7)
Prior hematuria/failed evacuation procedures ²	5 (20.8)
Pre-operative hospitalization (days) ¹	2.5 (1–4)

BMI, Body Mass Index; ASA, American Society of Anesthesiologists physical status classification; BPH, Benign Prostatic Hyperplasia; TURP, Transurethral Resection of the Prostate; PVP, Photoselective Vaporization of the Prostate, PAE, Prostate Artery Embolization, CBI, Continuous Bladder Irrigation

^1^median (interquartile range), ^2^N (%)

Baseline antithrombotic therapy was prevalent, with 45.8% (11/24) of patients on therapeutic anticoagulation and 37.5% (9/24) on antiplatelet therapy. Anticoagulation and antiplatelet therapy were held preoperatively when clinically feasible; they were both unable to be held in one patient, and antiplatelets alone were unable to be held in two additional patients due to absolute medical indications (e.g. recent cardiac stent placement). Upon admission, 33.3% (8/24) of patients presented with severe GH requiring pre-operative blood transfusions due to acute blood loss anemia. To manage bleeding prior to surgery, CBI was also utilized in 22 (91.7%) patients.

The median operative time for HoLEP was 102 min (IQR: 78–123.5 min), and remaining intraoperative characteristics are notated in Table 2. There were no intraoperative complications noted for any patient. The median length of stay following HoLEP was 2.5 days (IQR: 1-6.5, range: 1–37 days), with 41.7% (10/24) of patients being discharged on post-operative day one. Of note, one patient experienced a prolonged hospital stay of 37 days due to a non-urologic complication. The patient was otherwise clinically stable from a urologic standpoint and was pending discharge when a cardiac arrest occurred, suspected to be secondary to opioid overdose, with subsequent recovery following resuscitation. Bridging anticoagulation was not routinely utilized, reflecting the hemostatic advantages of HoLEP in high-risk patients. Anticoagulation and antiplatelet therapy were generally resumed during the index hospitalization, often at least one day prior to catheter removal and trial of void, to assess hemostatic stability after resumption of therapy and reduce concern for recurrent hematuria after catheter removal.

**Table 2:** Perioperative and post-operative outcomes

Characteristic	*N* = 24
Intra-operative clinical course	
Estimated blood loss (mL) ¹	100 (50–100)
Total energy (kJ) ¹	214.3 (183.5–279.8)
Procedure time (min) ¹	102 (78–123.5)
Enucleation time (min) ¹	44.5 (35.5–55.25)
Morcellation time (min) ¹	19.5 (14.5–39.25)
Specimen weight (g) ¹	156 (125.5–249)
Post-operative clinical course	
Post-operative length of stay (days) ¹	2.5 (1-6.5)
Total hospital length of stay (days) ¹	6 (3-10.25)
Post-operative Hemoglobin (g/dL) ¹	9.3 (7.6–10.6)
Received post-operative blood transfusion ²	7 (29.2)
Catheter and voiding outcomes	
POD catheter removed ¹	1 (1–2)
Passed initial trial of void ²	18 (75.0)
Discharged without Foley catheter ²	22 (91.7)
Catheter-free at last follow-up ²	24 (100)
Complications and follow-up	
30-day complications (CD I/II) ²	7 (29.2)
Readmission required (within 90 days) ²	4 (16.7)
Emergency Department visit required ²	5 (20.8)
Additional operative intervention required ²	0 (0)

POD, Post-operative Day, CD, Clavien-Dindo Grade

^1^median (interquartile range), ^2^N (%)

**Fig. 1:**
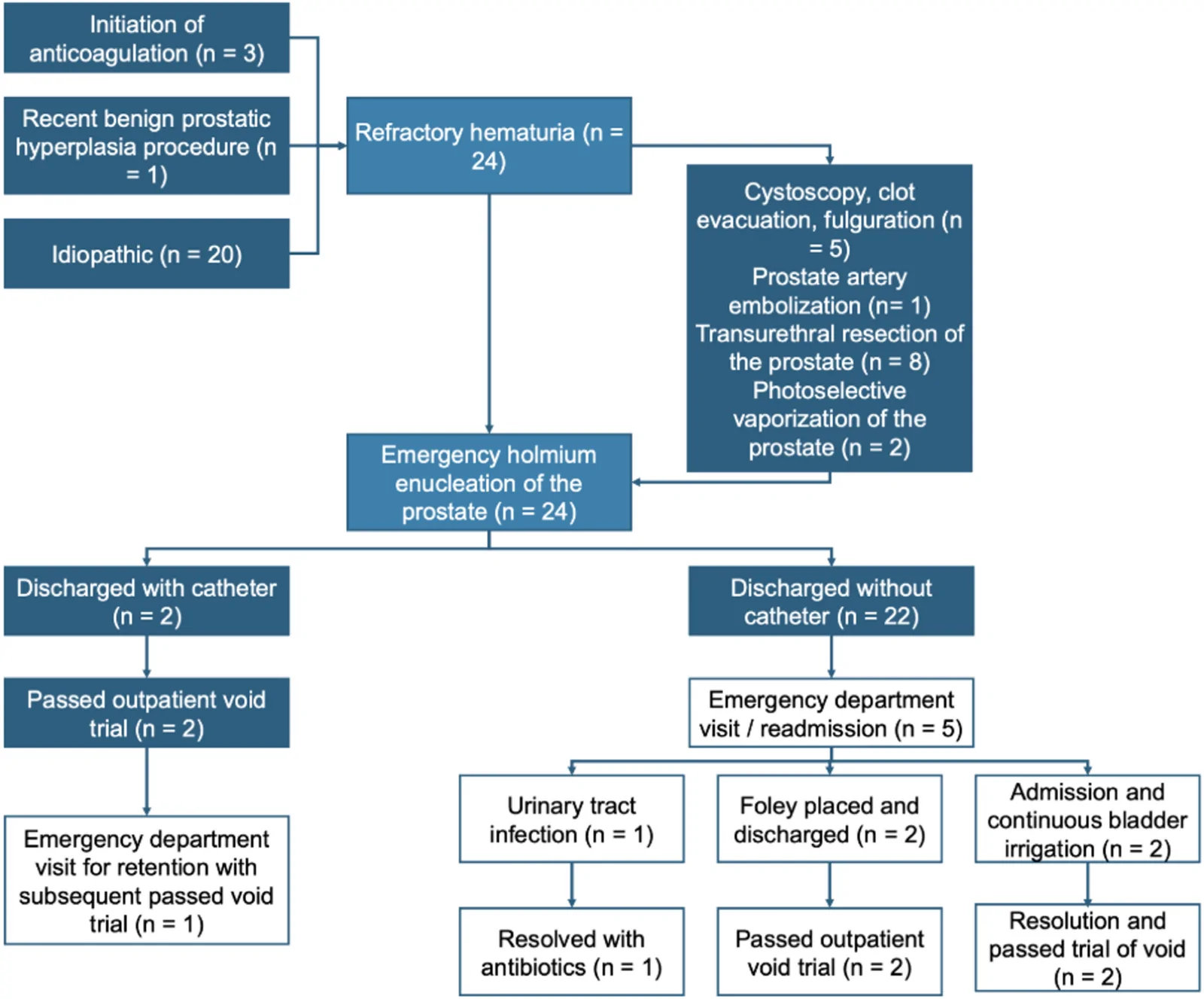
Flow chart depicting preoperative characteristics and postoperative clinical events among patients undergoing emergency HoLEP

Patients demonstrated excellent functional recovery; the median post-operative day for catheter removal was day 1. A complete flow chart of preoperative characteristics and postoperative events is presented in Fig. 1. The 30-day complication rate was 29.2% (7/24), with all events classified as minor Clavien-Dindo grade 1 or 2 complications. Post-discharge, 5 patients (20.8%) required an emergency department visit, and 4 patients (16.7%) required re-admission within 90 days. Readmissions were all related primarily to recurrent gross hematuria (16.7%). Specifically, two patients presented with hematuria complicated by catheter obstruction and significant hemoglobin decline, including one episode associated with syncope and another occurring in the setting of antithrombotic therapy requiring temporary cessation of aspirin and ticagrelor. Another patient was readmitted for acute urinary retention with hematuria and clot passage per urethra. The final readmission occurred following the resumption of anticoagulation, featuring recurrent hematuria complicated by a urinary tract infection requiring antibiotic therapy and Foley catheter placement. All readmitted patients were successfully managed with temporary re-catheterization and eventual catheter removal. No patients required additional re-operative interventions. As of the last follow-up, all 24 patients remain catheter-free and are actively voiding.

## Discussion

Severe GH often presents with urinary retention due to clot formation and requires temporization with catheterization, irrigation, and operative intervention such as cystoscopy, clot evacuation, and fulguration [4–6]. When the underlying etiology of GH is massive BPH, management can be challenging. Prior to HoLEP, our patients experienced extended hospital stays, high rates of failed conservative management with catheterization and continuous bladder irrigation, and previous attempts to manage their BPH. The clinical and surgical history of the patients in our study is consistent with literature emphasizing the challenging management of BPH-related GH [13]. Gross hematuria itself represents a major barrier to hospital discharge, as patients often remain admitted solely due to persistent visible bleeding despite otherwise stable clinical status. As such, failure to achieve rapid hemostasis prolongs hospitalization even in the absence of other medical indications for continued inpatient care. In our selected cohort, initial conservative and minimally invasive interventions did not provide sufficient control of bleeding to permit safe discharge or clinical stabilization. While adjunctive measures such as antifibrinolytics or hydrogen peroxide bladder irrigation have been described for disruption of intravesical clot burden and facilitation of clot evacuation, evidence remains limited and does not specifically address prostatic bleeding. Hydrogen peroxide irrigation was not utilized in our cohort.

Several studies have evaluated PAE for treating GH in the emergency setting. Tian et al. reported that on average, resolution of GH was 2.8 ± 1.1 days after PAE, and the average time catheters remained in place after the procedure was 4.8 ± 1.4 days after PAE [10]. At 3-month follow-up, 15% (3/20) of patients had recurrent hematuria and underwent TURP to completely resolve bleeding. In a review from Pereira and colleagues, treating refractory GH with PAE led to recurrence of hematuria after embolization in 15–28% of patients [5]. In several cases, repeat embolization was required to stop persistent bleeding. While PAE is minimally invasive and has been approved by the AUA for LUTS/BPH therapy, its use for refractory GH remains limited. In contrast, patients undergoing emergency HoLEP in our cohort experienced rapid and durable control of bleeding. The majority of patients achieved catheter removal on postoperative day 1, and gross hematuria resolved acutely in the postoperative course. While a small subset of patients experienced postoperative hematuria necessitating emergency department visit or readmission, often in the setting of anticoagulation resumption or catheter obstruction, these events were managed conservatively with temporary recatheterization and bladder irrigation. Further, all patients ultimately achieved catheter-free voiding without requiring repeat surgical intervention. Compared to PAE, which potentially leads to prolonged catheterization and higher recurrence rates, our findings suggest HoLEP superiority.

In the emergency setting, procedures such as TURP and PVP are unlikely to resolve refractory GH from massive prostatomegaly, as they do not completely remove the tissue such as in simple prostatectomy or HoLEP [4, 12]. Simple prostatectomy, which completely enucleates the bleeding prostatic tissue, however, has been associated with higher transfusion rates than HoLEP. Even after the reduction in blood loss and transfusion risk associated with robotic assistance compared with the open approach, available comparative data suggest that HoLEP has a favorable perioperative bleeding profile compared with RASP, including lower hemoglobin decline and reduced transfusion risk in meta-analytic evidence [14–16]. A study from Ibrahim et al. explored surgical outcomes of open simple prostatectomy for refractory hematuria due to BPH; they reported that the mean resected prostate volume was 127 g, with most patients requiring blood transfusion during initial presentation and perioperative management [17]. However, they did not specifically evaluate anticoagulation status as a determinant of open prostatectomy candidacy. Robotic simple prostatectomy has only been described in limited case-level evidence for refractory hematuria in this setting, with reported postoperative transfusion requirement, delayed catheter removal, and prolonged hospitalization [18]. Assmus et al. compared HoLEP to simple prostatectomy and found that HoLEP had shorter operative time, length of stay, catheter duration, and estimated blood loss [19]. Despite a larger mean prostate volume and higher baseline bleeding risk than reported in prior series, patients in our cohort experienced rapid resolution of hematuria, early catheter removal, and durable bleeding control without the need for re-operative intervention. While transfusions were required in a subset of patients—often preoperatively due to acute blood loss anemia—no patient required additional surgical or endovascular procedures for recurrent bleeding. These results portray the feasibility and safety of emergency HoLEP in a high-risk inpatient population and extend existing comparative data by demonstrating that the perioperative advantages of HoLEP over simple prostatectomy persist even in the acute, hemorrhagic setting.

A recent study from Elmansy and colleagues found that HoLEP was effective in managing GH that was not responsive to conservative management [4]. HoLEP was performed in 40 patients using conventional or MOSES™ laser technology (median resected weight: 81 g), with no statistically significant differences observed between modalities, although MOSES™ demonstrated numerically favorable intraoperative parameters in this small cohort. Of note, 95% of patients had a successful trial of void and catheter removal within 1 day of HoLEP. In contrast, our HoLEP cases were also performed with MOSES™ 2.0 laser technology on slightly larger prostates compared to Elmansy et al. and in a more emergent inpatient setting. We noted no intraoperative complications among our patients, regardless of anticoagulation status at the time of the procedure. Additionally, rates of catheterization improved after HoLEP: 91.7% of patients were discharged without a Foley catheter, and all 24 patients were catheter-free at last follow-up. Despite the larger prostates, emergency setting, and incidence of varied comorbidities and antiplatelet/anticoagulant usage, our patients did well after undergoing HoLEP and did not have any major complications.

Our study has several limitations. First, we did not compare our experience to controls who underwent HoLEP electively, as the purpose of this study was to describe the efficacy and safety of HoLEP in this novel setting. These patients had very large prostates and had a large set of comorbidities, including a high usage of anticoagulants and antiplatelets. Despite these factors, our patients did well with no major complications and are all now catheter independent. In addition, given the limited number of patients who present in this setting, we were unable to compare patients who underwent alternative treatments such as TURP and PAE for refractory GH secondary to massive BPH. However, individual patients received each of these treatment options, which proved unsuccessful at resolving GH and were remedied by this subsequent HoLEP procedure. Future studies should examine and compare these options prospectively to assist with decision-making for this complex clinical scenario. Additionally, HoLEP is not universally accessible and requires specialized equipment and institutional support, which may limit generalizability. The procedure also has a well-described learning curve, and outcomes may vary based on surgeon experience [20]. Prior work has demonstrated variability in HoLEP adoption across institutions, reflecting these barriers [20].

Currently, no dedicated guidelines specifically address management of refractory gross hematuria due to massive BPH in the acute inpatient setting. Based on our results, we support consideration of HoLEP after failure of non-operative methods; in our cohort, this was performed using MOSES™ laser technology, which may offer hemostatic and visualization advantages in the acute bleeding setting. For patients who cannot undergo HoLEP, such as those with cardiopulmonary contraindications or an inability to tolerate dorsal lithotomy position, PAE can be performed as an intervention for GH. With the recent approval of PAE by AUA for LUTS attributed to BPH, PAE is an appropriate intervention for treating refractory GH for patients who are not surgical candidates [6, 21]. However, with limited studies discussing definitive management, our investigation supports the safety and efficacy of HoLEP for refractory GH due to massive BPH in the emergency setting.

## Conclusion

In this single-center, single-surgeon cohort, HoLEP demonstrated favorable perioperative and functional outcomes for men with refractory GH due to massive BPH, including patients on ongoing anticoagulation. Patients experienced high rates of early catheter removal, durable catheter-free voiding, and no need for re-intervention. Treatment with HoLEP may offer a definitive intervention for the acute inpatient setting of recurrent gross hematuria. Especially in situations where conservative and endoscopic options fail, emergent HoLEP may hasten patient recovery and potentially reduce morbidity associated with treatment delay.

## Data Availability

No datasets were generated or analysed during the current study.
